# Antibiotic Resistance in the Drinking Water: Old and New Strategies to Remove Antibiotics, Resistant Bacteria, and Resistance Genes

**DOI:** 10.3390/ph15040393

**Published:** 2022-03-24

**Authors:** Ana Catarina Duarte, Sílvia Rodrigues, Andrea Afonso, António Nogueira, Paula Coutinho

**Affiliations:** 1 Centro de Potencial e Inovação em Recursos Naturais, Unidade de Investigação para o Desenvolvimento do Interior do Instituto Politécnico da Guarda (CPIRN-UDI/IPG), 6300-559 Guarda, Portugal; acduarte@ipg.pt (A.C.D.); smrodrigues91@gmail.com (S.R.); 2 CICS-UBI—Centro de Investigação em Ciências da Saúde, Universidade da Beira Interior, 6200-506 Covilhã, Portugal; 3 Laboratório de Saúde Pública da Guarda, Unidade Local de Saúde da Guarda (ULSGuarda), Avenida Rainha Dona Amélia, 6300-858 Guarda, Portugal; 4 Laboratório de Saúde Pública de Bragança, Unidade Local de Saúde do Nordeste (ULSNE), Rua Eng. Adelino Amaro da Costa, 5300-146 Bragança, Portugal; andrea@ipb.pt; 5 Centro de Investigação de Montanha (CIMO), Instituto Politécnico de Bragança (IPB), Campus de Santa Apolónia, 5300-253 Bragança, Portugal; ajmnogueira@ipb.pt

**Keywords:** antibiotic resistance, drinking water, water treatment, antibiotic resistance genes, nanotechnology, microalgae

## Abstract

Bacterial resistance is a naturally occurring process. However, bacterial antibiotic resistance has emerged as a major public health problem in recent years. The accumulation of antibiotics in the environment, including in wastewaters and drinking water, has contributed to the development of antibiotic resistant bacteria and the dissemination of antibiotic resistance genes (ARGs). Such can be justified by the growing consumption of antibiotics and their inadequate elimination. The conventional water treatments are ineffective in promoting the complete elimination of antibiotics and bacteria, mainly in removing ARGs. Therefore, ARGs can be horizontally transferred to other microorganisms within the aquatic environment, thus promoting the dissemination of antibiotic resistance. In this review, we discuss the efficiency of conventional water treatment processes in removing agents that can spread/stimulate the development of antibiotic resistance and the promising strategies for water remediation, mainly those based on nanotechnology and microalgae. Despite the potential of some of these approaches, the elimination of ARGs remains a challenge that requires further research. Moreover, the development of new processes must avoid the release of new contaminants for the environment, such as the chemicals resulting from nanomaterials synthesis, and consider the utilization of green and eco-friendly alternatives such as biogenic nanomaterials and microalgae-based technologies.

## 1. Introduction

Bacterial antibiotic resistance is a major public health problem. Worldwide, about 700,000 deaths occur annually due to infections caused by resistant bacteria. In 2050, it is expected that this number will increase, reaching 10 million cases per year if no preventive measures are universally adopted [1]. In parallel, antibiotic resistance genes (ARG) and antibiotic resistant bacteria (ARB) can have an environmental distribution, which is why they have been recognized as emergent environmental pollutants [2].

The dissemination of ARB and ARG in the environment results from the inappropriate use of antibiotics in human and veterinary clinics, the incorrect elimination of expired antibiotics, the increase in discharges of pharmaceutical industrial wastewaters, and the reduced efficiency of wastewater and drinking water treatment plants [3,4]. Indeed, reducing antibiotics use per se is insufficient to control the environmental dissemination of ARBs and ARGs in drinking water [5,6,7].

The efficiency of water treatment processes plays a critical role in disseminating antibiotic resistance throughout the water distribution systems [2]. The selection of the processes used in drinking water treatment plants is based on the physicochemical characteristics of the water. Coagulation/flocculation, sedimentation, filtration, and disinfection are the most common processes used worldwide [8,9]. However, monitoring ARB and ARG prevalence after the treatments is usually neglected [7]. Moreover, recent technologies developed for water remediation, such as advanced oxidation, biological and granular activated carbon filtration, and membrane filtration, also fail to remove ARGs or even contribute to their dissemination along the water distribution systems [10,11].

Therefore, developing accessible and efficient water remediation processes to remove antibiotics, ARB, and especially ARGs remains a challenge.

Among the strategies developed in recent years, nanotechnology and microalgae-based technologies have shown great potential to address some limitations of the current water treatment processes. In addition, future studies should focus on the potential use of these technologies to control the dissemination of ARB and ARG in drinking water distribution systems. This literature review aims to discuss the limitations of conventional and advanced water treatment processes in addressing the dissemination of antibiotic resistance. On the other hand, we summarized nanotechnology and microalgae-based technologies recently reported to address antibiotic resistance in wastewaters and drinking water.

## 2. Antibiotic Resistance

Antibiotics are indispensable for treating infectious diseases in humans and animals, and their use in the last century allowed to improve healthcare services and increase life expectancy [12]. However, the inappropriate consumption and the incorrect disposal of antibiotics favored its accumulation in the environment, including in raw and treated drinking water [9]. The antibiotic’s presence in the environment produces a selective pressure leading to ARB and ARG dissemination. Bacterial infections are harder to treat, as commonly used antibiotics are less effective or even ineffective, which might cause uncurable infections and will undoubtedly impact medical costs and mortality numbers worldwide [13]. For this reason, antibiotic resistance has become, in recent decades, a major health concern. Several bacterial species can cause infection in humans. However, *Escherichia coli* and multidrug-resistant ESKAPE bacteria (*Enterococcus faecium*, *Staphylococcus aureus*, *Klebsiella pneumoniae*, *Acinetobacter baumannii*, *Pseudomonas aeruginosa*, and *Enterobacter* species) pathogens [14,15] were, in 2017, included in the World Health Organization report, classified as critical for public health, and listed as high priority ARB [16].

### 2.1. Bacteria Mechanisms to Antibiotic Resistance

Bacterial species can display antibiotic resistance by intrinsic or acquired mechanisms that prevent antibiotics access to their bacterial targets or result in antibiotic inactivation [17,18,19].

#### 2.1.1. Intrinsic Antibiotic Resistance

Intrinsic resistance is related to inherent structural or functional properties shared within a bacterial species independently of previous antibiotic exposure [19]. For example, antibiotics must cross the bacterial cell wall to reach their intracellular target. Gram-negative bacteria are intrinsically less permeable than Gram-positive due to their outer membrane, which acts as a permeability barrier [17]. Vancomycin antibiotic inhibits peptidoglycan crosslinking, and is effective in Gram-positive bacteria, but not in Gram-negative bacteria as it cannot cross the outer membrane. Despite this, antibiotics can enter the cells in Gram-negative bacteria by diffusion via porin proteins located in the outer membrane. Bacteria can express different drug efflux pumps such as ATP (adenosine triphosphate)-Binding Cassette (ABC) superfamily, Resistance Nodulation Division family, Multidrug and Toxin Extrusion superfamily, Major Facilitator Superfamily, and the Small Multidrug Resistance family, which can remove antibiotics from the cell cytoplasm to the extracellular environment [19]. For example, *S. aureus* expresses the efflux pump *NorA* that is responsible for fluoroquinolones efflux [20].

#### 2.1.2. Acquired Antibiotic Resistance

**Figure 1:**
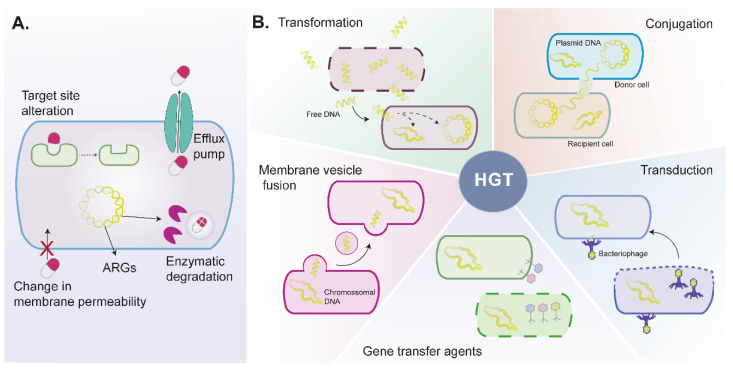
Schematic representation of antibiotic resistance mechanisms in bacteria. (**A**). Bacteria might present or acquire mechanisms to disable antibiotics, including alterations on target site, decreased permeability that impairs antibiotics cellular uptake, expression of drug efflux pumps that remove antibiotics from cells, and production of enzymes that modify or degrade antibiotics. (**B**). Horizontal gene transfer allows the exchange of genetic information between the same or different species by transformation, conjugation, membrane vesicle fusion, transduction, and gene transfer agents mechanisms. ARGs—antibiotic resistance genes, HGT—horizontal gene transfer.

In addition to intrinsic resistance mechanisms, bacteria can also acquire antibiotic resistance mechanisms, including decreased cell permeability, increased expression of efflux pumps, modification of antibiotic targets (by genetic mutation or post-translational modification of the target), and antibiotic enzymatic inhibition or degradation (Figure 1A) [17,19,21]. To limit antibiotics’ access to cellular targets, bacteria might reduce cell permeability by altering porin proteins expression and/or function [19]. Overexpression of efflux pumps contributes to increased antibiotic extrusion, leading to low intracellular accumulation. The *norA* gene is found overexpressed in several strains of *S. aureus*, especially in methicillin-resistant strains (MRSA), and is associated with acquired resistance to fluoroquinolones such as ciprofloxacin [22,23]. Additionally, mutations codifying for changes in the antibiotic target are an important resistance mechanism against fluoroquinolones antibiotics which target the GyrA and ParC/GrlA proteins in Gram-negative and Gram-positive bacteria, respectively [24]. Bacteria can also present target changes, such as those observed in *S. aureus* MRSA strains, which result from acquiring the *mecA* gene and confers resistance against β-lactams [25]. The β-lactams action is based on its ability to inhibit penicillin binding-proteins at the bacteria cell wall. However, the cell wall of MRSA strains presents PBP2a, which is encoded by the gene *mecA* and is a penicillin-binding protein with low affinity to β-lactams.

The mechanisms mentioned can be acquired through spontaneous mutation and, more frequently, by transference of genetic material from a foreign source or horizontal gene transfer [19].

##### Horizontal Gene Transfer

Horizontal gene transfer of ARGs is probably the major factor contributing to the occurrence of new bacterial resistant strains [26]. The horizontal gene transfer mechanisms involve transformation, conjugation, transduction, membrane vesicle fusion, and gene transfer agents (Figure 1B) [19,27].

Transformation traduces the uptake of free DNA from a foreign competent bacteria that can be incorporated into the recipient bacteria after the death and lysis of a bacteria. In conjugation, cell–cell contact between bacteria allows the exchange of plasmids from a donor to a recipient cell. Plasmidic DNA replicates independently from the DNA chromosomic and can carry other mobile genetic elements (e.g., transposons and integrons) and several ARGs [19]. Transduction is a process mediated by bacteriophages. Bacteriophages infect bacteria, and after bacteria lysis, they can incorporate DNA fragments that will be transferred to another bacteria genome [27,28]. Bacterial membrane vesicles carry lipids, proteins, and DNA that can be released to the external environment under stressful conditions, fuse, and transform other bacteria [29]. Recently, Lee and colleagues showed that membrane vesicle fusion could transfer β-lactam resistance substances from *S. aureus* (MRSA strain) to *E. coli*, resulting in increased β-lactamase activity and conferring resistance to β-lactam antibiotics [30].

Gene transfer agents are phage-like particles that carry random DNA fragments, lacking DNA encoding machinery and self-propagating ability [31]. Gene transfer agents are produced by bacteria and released after bacteria lysis. These agents present some advantages over natural transformation and conjugation, as they can protect DNA from environmental factors and are not limited by cell-to-cell contact.

#### 2.1.3. Biofilm Antimicrobial Resistance

Biofilm antimicrobial resistance comprises both innate and acquired mechanisms [32,33]. Biofilms are aggregates of microorganisms surrounded by a self-produced matrix of extracellular polymeric substances, attached to natural or artificial surfaces resulting from the adaptation to environmental stressors, such as antibiotics, and promote bacteria growth and survival [27,33].

Innate mechanisms of biofilms include the presence of a barrier established by the matrix of extracellular polymeric substances that limits antibiotic diffusion through the biofilm [32,33]. Additionally, hypoxic zones in deeper parts of the biofilms slow bacteria growth and enable their tolerance against antibiotics that target metabolic processes. In accordance, the efficacy of some antibiotics such as cephalothin and vancomycin is reduced in older biofilms [34]. Besides structural organization and stability conferred by the extracellular polymeric matrix, biofilms display resource capture by sorption, digestive capability, intercellular communication, and enhanced metabolic activity that promotes bacteria survival [33].

The high cell density, heterogenous bacteria population, and accumulation of mobile genetic elements enhance the horizontal gene transfer processes (transformation, conjugation, transduction, vesicle fusion, and gene transfer agents), enabling the transfer and acquisition of ARGs between the different bacteria present in the biofilm [27,33]. Moreover, the matrix of biofilms confers higher physical stability, facilitating and improving plasmid conjugation that requires cell-to-cell contact than free-living bacteria [35,36]. Different microorganisms can be associated within a biofilm allowing horizontal gene transfer and metabolic interactions, thus promoting biofilm dispersion and higher ARB dissemination in the environment [27]. Biofilms are commonly found in water distribution systems, representing a high risk for human health, and should be considered as one of the most relevant factors to be controlled in water treatment plants and water distribution systems [37].

## 3. Critical Factors for Antibiotic Resistance Widespread in Environment and Water Sources

In recent years, emergent evidence reinforces antibiotic resistance as a major human health problem with antibiotics, ARB, and ARG being systematically detected in diverse environmental matrices such as air, soil, groundwater, surface water, wastewater, and drinking water [2]. The main factors identified to contribute to this environmental widespread are (i) human and veterinarian inappropriate antibiotic consumption; (ii) farm and aquaculture activities; (iii) health care and pharmaceutical facilities discharge; and (iv) inefficient removal of antibiotics at water treatment plants [2,38,39,40].

### 3.1. Human and Veterinary Consumption

In recent decades, antibiotic consumption has risen due to human population increase, improved life quality, easier access to medicines, and overall improved health care services. Human population increase drove higher demand for animal protein and the intensification of food production [41,42].

The antibiotic consumption between 2000 and 2015 was analyzed in 76 countries, including high- and low-income countries, by Klein and colleagues [38]. Overall antibiotic consumption rate grew by 39%, with low- and middle-income countries showing the greatest increase, although antibiotic use was found to be higher in high-income countries. More recently, an analysis of antibiotic consumption by EE/EEA (European economic area) countries between 1997 and 2017 showed that β-lactams antibiotics (penicillin) remained the most consumed over the years [43]. Furthermore, the overall antibiotic consumption remained unaltered. Despite sulfonamides and trimethoprim consumption decreasing in most countries, the consumption of other antibiotics increased. The same conclusions were described in the latest report from European Centre for Disease Prevention and Control, which analyzed antibiotic consumption from 2010 and 2019 and did not notice significant differences over time [44]. These studies indicate that, despite European countries’ measures to increase awareness regarding antibiotic use and prevent antibiotic resistance dissemination, additional efforts are still required to reduce global human antibiotic consumption.

On the other hand, antibiotics are widely used in veterinary activities, farms, and aquaculture [13,40,45,46,47]. In aquaculture, antibiotics are added directly to water as a preventive measure (prophylaxis). However, aquaculture systems are rich in diverse bacteria, which favor horizontal gene transfer and, thus, the dissemination of ARG and ARB in the aquatic environment [39,48].

The antibiotics and metabolites are excreted by humans and animals through urine and feces, reaching sewage systems, as unchanged or as conjugations of glucuronic and sulfuric acid [46,49], thus contributing to water contamination. Therefore, it is necessary to be aware of the correct use of antibiotics to restrain antibiotic resistance widespread. It is expected that reducing antibiotics consumption would decrease the levels of antibiotics found in wastewater and drinking water.

### 3.2. Health Care Facilities and the Pharmaceutical Industry

Health care facilities are known to be a hotspot of ARB, and usually, their wastewaters are discharged without appropriate pretreatment into the sewage system [50]. Hospital wastewaters display a higher ecotoxicity risk due to the high levels of pharmaceuticals such as antibiotics, ARB, and ARGs [51,52,53]. Even though the wastewater treatment plants can remove most ARB, ARG elimination is more challenging. Consequently, high levels of ARGs and mobile genetic elements have been detected in the effluents of hospital wastewater treatment plants [51,53,54,55]. Additionally, most of the ARG detected in effluents are associated with resistance to antibiotics clinically relevant, such as β-lactams, sulfonamides, macrolides, and tetracyclines [52,54].

Similarly, effluents of the pharmaceutical industry or pharmaceutical wastewater treatment plants showed high antibiotic levels [12]. This provides high selection pressure that favors the proliferation of ARG and mobile genetic elements [56,57,58], which is responsible for their persistence in the downstream effluents [59]. So, the total elimination of antibiotics is necessary to control ARG abundance in effluents [58]. Therefore, it is urgent to establish special regulatory measures to treat industrial wastewaters to limit antibiotic resistance proliferation in the aquatic environment.

### 3.3. Biofilm Formation in Drinking Water Distribution Systems

Drinking water distribution systems comprise a network of extensive pipelines that deliver potable water from drinking water treatment plants to consumers. In addition, water reservoirs that allow long term water storage are important drinking water resources for the human population. However, these structures commonly harbor a great biofilm area, detaching and releasing ARBs and ARGs, causing drinking water contamination [9,10,60,61,62,63,64]. Therefore, the development of biofilms in drinking water distribution systems and inefficient water treatments are a huge risk for human health [37,63]. In accordance, previous epidemics outbreaks have been associated with contaminated drinking water [37].

To date, several reports have elucidated the risk of biofilm formation in drinking water distribution systems concerning antibiotic resistance [10,60,61,63,64,65,66]. High-throughput quantitative PCR allowed the detection of 285 ARGs and other mobile genetic elements in water samples from two drinking water treatment plants [10]. This work showed that biological activated carbon water treatment leads to ARGs increase. Additionally, the chlorination at the final water treatment step enhanced the relative abundance of ARGs. ARGs were analyzed across water distribution systems and in tap water. In tap water, ARGs’ absolute abundance, especially β-lactam resistance genes, was found to increase 6.4- to 109.2-fold compared to finished water, showing that pipeline transportation contributes to antibiotic resistance dissemination [10].

Furthermore, the detection and enrichment of mobile genetic elements, such as transposases and *intI-1* genes, also suggest that they play a critical role in antibiotic resistance dissemination in drinking water. In accordance, Chan and colleagues found that 58% of the bacteria detected in the distributed water was released from the pipe biofilm [61]. More recently, sediment samples from 10 water reservoirs showed the presence of 174 ARGs, being the most prevalent the multidrug-, sulfonamide-, and vancomycin-ARGs [60]. The mobile genetic elements were identified as the main biotic factors contributing to ARGs dissemination in the analyzed sediments.

Overall, biofilm formation in drinking water distribution systems aggravates antibiotic resistance dissemination in drinking water, affecting its quality and safety. Therefore, it is necessary to develop new water remediation systems to efficiently remove ARGs at the drinking water treatment plants, inhibit biofilm formation, and eliminate the ARGs and ARBs in pipes and water reservoirs.

### 3.4. Inefficient Antibiotic, ARB, and ARB Removal by Water Treatment Processes

Worldwide, since the early 20th century, a combination of coagulation, sedimentation, and filtration has been applied in water treatment. Conventional water treatment plants use a combination of coagulation, flocculation, sedimentation, filtration, and disinfection units, to provide clean and safe drinking water to the public. However, these technologies present several drawbacks. Most wastewater and drinking water treatment processes require the use of hazardous chemicals, achieve low removal efficiency of contaminants, formation of by-products, and have high costs, among others [67]. While conventional water treatments effectively eliminate bacteria, the removal of ARG by processes such as coagulation, sedimentation/clarification, sand filtration, and biological activated carbon filtration might not be achieved. Indeed, these processes produced contradictory results concerning the ARG removal efficiency of different water treatment processes (Table 1).

Some studies demonstrated that the conventional treatments including sand filtration and chlorination can achieve good performance in the removal of some ARGs, especially of sulfonamides resistance genes *sul1*, *sul2*, and *sul3* [68,70,72,73,76], aminoglycosides ARGs *aadA*, *aadB*, and *aadE* [68,69], β-lactams ARGs *bla_OXA-1_* and *bla_TEM_* [70], macrolides ARGs *ereA*, *ermF*, *ermG*, *ermX*, *mefA*, and *mphA* [69,72], quinolones ARGs *qepA*, *qnrA*, *qnrD*, and *qnrS* [70,73], and tetracyclines ARGs *tetA*, *tetC*, *tetG*, *tetO*, *tetQ*, *tetS*, and *tetX* [69,70,72,73] (Table 1). However, the removal efficiency of other ARGs such as *aphA1*, *strA*, *strB*, *ermB*, *qnrB*, and *tetM* showed less consistent results, with reports showing both decreases and increases in their expression [69,70,73]. Moreover, conventional water treatments were ineffective in the removal of efflux pump resistance genes *mexF*, *mexT*, and *mexW*, macrolide ARG *ermC*, and tetracycline ARG *tetB* [68,70].

Besides chlorination, ozonation and ultraviolet (UV) radiation are two other common disinfection technologies applied in wastewater and drinking water treatment plants and are dependent on the dose used and other physicochemical factors [80]. A process involving low-pressure UV was recently recommended as a supplementary bactericidal treatment to remove ARG in terminal water treatment [77]. The combination of UV treatment with conventional processes used in the water treatment system resulted in a higher reduction in ARB [81] and gene inactivation, and higher removal rates of *bla_TEM1_*, *mphA*, and *tetA* [72,79]. However, UV radiation increased the abundance of *aadA*, *mexF*, *mexT*, *mexW*, *bacA*, and *sul1* resistance genes [68]. A previous study showed that UV LED was able to eliminate ARB and ARG (*tetL*), but resistance has reemerged naturally after disinfection [79]. Therefore, the UV/UV LED radiation process for water treatment to ARB and ARG removal is a promising strategy but requires further optimization.

In recent years, different technologies have emerged for the management of drinking water systems, such as biological activated carbon and membrane filtration technology [2,82].

Several reports have shown that biological activated carbon increases the abundance of ARGs (Table 1) [10,11,69,83,84]. This has been associated with the adhesion of bacteria to the filter surface, formation of biofilms, and consequent horizontal gene transfer [83,85]. Similar results were obtained for granular activated carbon filters [71,73]. The filtration by powder activated carbon seems to be more competent in removing the ARG [73]. 

Membrane filtration is another advanced water treatment process that includes ultrafiltration, nanofiltration, osmosis reverse, and forward osmosis [2,82]. The membranes act as physical barriers to limit the passage of pathogens and other water contaminants during the water treatment. 

Reverse osmosis filtration proved to reduce the absolute abundance of sulfonamide (*sul1* and *sul2*), tetracycline (*tetB*, *tetG*, and *tetX*), macrolides (*ermF*), and quinolones (*qnrA*, *qnrB*, and *qnrS*) resistance genes [74]. Liang and colleagues [75] showed a reduction of about 99% of the total ARGs by membrane filtration that included a step of ultrafiltration and a two-stage reverse osmosis process. Overall, 16 types of ARGs were analyzed, including *tetQ*, *tetM*, *tetW*, *sul1*, *sul2*, *ermF*, and *cfrA*. However, despite the significant reduction in their absolute abundance, ARGs remained at detectable levels in final water, as well as 16S rRNA that only showed a slight decrease. These results are in accordance with previous data showing that membrane technology enhances the removal of bacteria and of some ARGs in comparison to conventional water treatment processes [86]. However, ARGs are not totally removed from water samples by membrane filtration, and performance varies across different studies (Table 1) [74,75,78,86]. Le and colleagues showed that microfiltration was able to completely remove ARB and reduce some ARGs, but *bla_KPC_*, *bla_NDM_*, *bla_SHV_*, *ermB*, *intI1*, *sul1*, and *tetO* persisted after the treatment [86]. Another study noticed an increase in sulfonamides (*sul1* and *sul2*) and tetracyclines-resistance genes (*tetA*, *tetB*, *tetM*, *tetO*, and *tetX*) after ultrafiltration [78].

In addition, membrane technology presents a major drawback: the risk of biofouling [82,87,88]. Microorganisms can adhere to the surface of the membranes, forming biofilms, thus supporting ARB proliferation. Diverse bacterial species, including *Klebsiella* sp., *Staphylococcus* sp., and *E. coli*, have been found in the biofouling of filtration membranes [87]. Additionally, chlorination pretreatment, used to prevent membrane fouling, increases the risk of ARGs in the reverse osmosis process [88]. Other approaches to reduce the risk of fouling involve membrane surface modification or membrane cleaning, which increases the associated costs.

Moreover, parameters such as molecular weight cut off and surface charge of membranes must be optimized to improve ARGs removal. A recent study demonstrated that membranes with a cut off smaller than 5000 Da and positively charged, contrarily to negatively charged DNA, enhance free DNA retention and adsorption [89].

Overall, membrane technology for water treatment and ARGs removal presented promising results but is still insufficient. Additionally, ARGs can be retained at the membranes during ultrafiltration, nanofiltration, or osmosis reverse processes but are not biodegraded. Thus, ARGs entrapped in the membranes can be released to the water in case of membranes breach or damage.

Therefore, the inefficient performance of conventional and advanced water treatment processes justifies the need for more efficient and affordable remediation systems that remove emergent pollutants, including antibiotics, ARB, and ARG, from the environment and water sources.

## 4. Promising Strategies to Reduce Antibiotic Resistance

The mitigation of antibiotic resistance dissemination in the environment should include strategies targeting the correct elimination of (i) antibiotics in the wastewater and drinking water treatment plants, (ii) ARBs in wastewater and drinking water treatment plants and biofilm development in drinking water distribution systems, and (iii) ARGs in both drinking water treatment plants and distribution systems. In recent years, several studies have reported new strategies for water remediation, including adsorption and degradation of pollutants based on biomaterials, nanomaterials, and microalgae. The following sections will discuss the removal efficiency of contaminants such as antibiotics, ARBs, and ARGs by these systems and the mechanisms involved.

### 4.1. New Approaches for Antibiotics Elimination

Despite the current processes used in wastewater and drinking water treatment plants to reduce antibiotic levels, antibiotics are still detected in drinking water [90,91]. Importantly, antibiotic accumulation imposes a high selective pressure in the environment, facilitating bacterial acquisition of resistance mechanisms [92]. A recent study showed that the presence of sulfadiazine and ciprofloxacin induced the enrichment of total bacteria and ARGs in drinking water from distribution systems compared to raw water [93]. Additionally, bacteria displayed enhanced enzymatic activities and extracellular polymeric substances production, promoting biofilm formation in the surfaces of the pipelines. Thus, antibiotics’ efficient removal in drinking water treatment plants presents a huge role in controlling antibiotic resistance dissemination. Recent strategies to overcome this issue include adsorption and degradation of antibiotics using nanotechnology and microalgae-based technologies (Table 2).

#### 4.1.1. Antibiotics Adsorption and Degradation Using Nanotechnology

Nanotechnology presents many applications, and several studies have analyzed its ability to counteract antibiotic resistance. Different nanomaterials, including nanoparticles (NPs), nanocomposites, nanotubes, and others, have been engineered to promote antibiotics removal (Table 2). Among the most promising nanomaterials, we found bimetallic and biogenic NPs as well as composites.

Bimetallic NPs have shown excellent results regarding antibiotics removal [107,110]. Ni/Fe NPs removed 97.4% of tetracycline through both adsorption and degradation mechanisms [110]. In another study, Cu (core) and Fe_3_O_4_ (shell) were combined to synthesize bimetallic NPs and showed great ability to remove oxytetracycline (>99%), improving Cu and Fe_3_O_4_ single catalytic activity [107]. Moreover, Cu@Fe_3_O_4_ presented good reusability potential, removing 97% of oxytetracycline after five cycles. However, the production of these materials is associated with the excessive use of hazardous chemicals that can produce new pollutants and present toxicity to the environment [126]. So, this process should be monitored considering the ratio of risk/benefit.

In this sense, biogenic nanomaterials emerged as a green alternative to conventional approaches to water treatment, being more eco-friendly, safer, and cost-effective. Biogenic nanomaterials, including NPs, nanorods, nanowires, and nanotubes, can be synthesized from different microorganisms (e.g., bacteria, fungi, and algae) and plants or bio-waste products instead of synthetic chemicals. These nanomaterials act as biocatalysts and adsorbents, contributing to the removal of toxic compounds such as heavy metals, hazardous dyes, pesticides, and pharmaceutical pollutants from water [126,127,128]. However, only a few studies analyzed its application and performance in the removal of antibiotics from water [102,103,129]. Biogenic platinum and palladium NPs synthesized from *Desulfovibrio vulgaris* bacteria allowed the remotion of ciprofloxacin and sulfamethoxazole [129]. Bio-platinum NPs showed higher catalytic activity and promoted the removal of 70% and 85% of ciprofloxacin and sulfamethoxazole, respectively. However, the reusability of Bio-platinum NPs was only ensured for three cycles. Another study that used bimetallic nZVI-Cu NPs synthesized from pomegranate rind extract showed a reduction of 72% of tetracycline [103]. Furthermore, bentonite addition enhanced NPs stability, allowing the removal of 95% of tetracycline, but, like Bio-platinum NPs, presented low reusability.

Although green synthesis of nanomaterials is a sustainable solution that should be exploited in the future for water treatment, some issues, such as biogenic NPs yield, stability, size, aggregation, reusability, and fabrication costs, are still unsolved [126,127].

Despite this, nanotechnology holds promise to remove antibiotics efficiently from water treatment plants. Moreover, nanomaterials’ application in membrane filtration technology has been shown to improve antibiotics removal [130] and inhibit biofouling [131,132]. Thin-film nanofiber membranes with UiO-66 NPs were fabricated and used in the forward osmosis process to evaluate antibiotics rejection. Membranes functionalized with the NPs increased the water flux and the rejection rate, above 99.9%, of six antibiotics (sulfamethoxazole, sulfamethazine, trimethoprim, erythromycin, chloramphenicol, and tetracycline) [130].

#### 4.1.2. Bioadsorption, Bioaccumulation, and Biodegradation of Antibiotics by Microalgae-Based Technologies

Microalgae-based technology is an eco-friendly strategy suitable for water remediation applications. In recent years, many studies have demonstrated microalgae-based technologies’ potential and efficiency in the removal of antibiotics (Table 2). The main mechanisms used by microalgae for antibiotic removal include bioadsorption, bioaccumulation, and biodegradation [133,134,135].

Bioadsorption of antibiotics can occur in the microalgae cell membrane or into organic substances excreted by microalgae, such as exopolysaccharides. Microalgae bioadsorption capability depends on the microalgae species and their physical and chemical properties, including surface chemistry, surface area, and target antibiotic structure [133]. Chen and colleagues compared the ability of *Chlorella vulgaris* and *Chrysosporum ovalisporum* to remove sulfadiazine, sulfamethazine, enrofloxacin, and norfloxacin during 16 days [113]. Overall, *C. vulgaris* showed better performance, but neither microalgae could efficiently remove norfloxacin. Furthermore, microalgae capacity to remove antibiotics depends on antibiotic concentration and decreases with higher antibiotic concentration. However, desorption of antibiotics was observed on day 11 or day 16. Besides living microalgae cells, the bioadsorption of antibiotics by microalgae biomass was achieved for the removal of tetracycline [136]. So, microalgae have a great potential to remove antibiotics from water by bioadsorption, but this is not the most suitable process to efficiently remove antibiotics, as it can be reversible. Thus, antibiotics might be rereleased to the environment.

Bioaccumulation, contrarily to bioadsorption, is an active metabolic process that comprehends the uptake of antibiotics by living microalgae cells and is affected by several factors, such as temperature, pH, contact time, and antibiotic concentration [133]. Bioaccumulation can be seen as an intermediate step between bioadsorption (accumulation of antibiotics on the cell membrane) and biodegradation (intracellular degradation of antibiotics). Biodegradation is the most effective mechanism for antibiotics removal, also for being an irreversible process that can result in less toxic by-products.

As biodegradation depends on the cellular metabolism of microalgae, antibiotics removal efficiency differs among microalgae species. *Chlorella pyrenoidosa* and *Microcystis aeruginosa* abilities to remove cefradine and amoxicillin by biodegradation were analyzed [111]. *C. pyrenoidosa* was more efficient in the removal of the antibiotics, removing about 42% and 71% of cefradine and amoxicillin, respectively. Additionally, it showed higher tolerance to both antibiotics compared to *M. aeruginosa*. In another study, tetracycline removal was successfully obtained with *M. aeruginosa* and *C. pyrenoidosa*. *M. aeruginosa* showed a faster and more efficient removal of tetracycline than *C. pyrenoidosa*, about 99% within 2 days due to adsorption, bioaccumulation, and biodegradation mechanisms. On the other hand, *C. pyrenoidosa* contribution to tetracycline removal was achieved mainly by abiotic photolysis, hydrolysis, and cation-binding [122]. In another study, ciprofloxacin and sulfadiazine removal by *Chlamydomonas* sp. Tai-03 occurred mainly through biodegradation (65.05%) and photolysis (35.60%) [123].

Overall, microalgae-based processes show great potential to be applied in water remediation to remove antibiotics, which are responsible for the selective pressure on ARB and consequent ARG dissemination.

##### Microalgae-Bacterial Consortium

Some recent reports support the use of microalgae-bacterial consortiums for water remediation [67,124,125]. A microalgae-bacterial consortium, where *Chorella sorokiniana* was the predominant microalgae species, was able to remove about 54% of sulfamethoxazole from wastewater treatment plant effluents, mainly through biodegradation by bacteria. This resulted from a symbiotic action of bacteria and microalgae that act as an oxygen source for bacteria development [125]. In another study, the microalgae-bacterial consortium showed great capacity (>90%) to remove two other antibiotics, cephalexin and erythromycin, through biodegradation from wastewater treatment plant effluent [124]. In addition, a microalgae-bacterial consortium is a promising approach for the biodegradation of pharmaceutical compounds such as antibiotics compared to pure microalgae cultures [137,138]. Recently, Wang and colleagues reported enhanced chlortetracycline removal by microalgae-bacteria compared to pure microalgae, most probably due to the increasing microalgae tolerance to high concentrations of the antibiotic [67]. Moreover, the microalgae-bacterial consortium removed about 80% of chlortetracycline, at high concentrations (80 mg/L), primarily via bioadsorption, followed by biodegradation mediated by extracellular enzymatic action. Thus, these results support the microalgae-bacterial consortium as a promising strategy to eliminate antibiotics in wastewater and drinking water treatment plant facilities. However, their performance regarding ARB and ARGs occurrence must also be analyzed.

### 4.2. New Approaches for ARBs and ARGs Elimination

Until recently, the lack of appropriated molecular tools to evaluate the occurrence of ARB and ARG in drinking water sources constrained their risk assessment. The development of new techniques such as high-throughput quantitative PCR, metagenomics, and whole-genome sequencing [139], have contributed to analyzing the occurrence and dissemination of several ARBs and ARGs in wastewater and drinking water [60,140,141,142]. The systematic detection of ARB and ARG, even after water treatment, has raised several warnings regarding the safety of drinking water and the risks to human health. Moreover, as previously discussed, most of the water treatment processes usually applied are inefficient on ARG removal, and thus new methods must be exploited.

#### 4.2.1. ARBs and ARGs Removal by Nanotechnology

There are already some reports describing new approaches to ARBs and ARGs elimination, based on nanomaterials with great adsorption potential, such as metallic NPs [143,144,145,146,147,148,149,150], electrocatalytic tools such as carbon nanotubes [151], and microalgae [119,124,125] (Table 3).

Duan and colleagues compared the efficiency of single metal nanoscale iron particles (NIPs), bimetallic (NICPs), and single and bimetallic NIPs modified with Ginkgo biloba L. (GNIPs and GNICPs, respectively) in bacteria and ARG removal [147]. Overall, bimetallic NIPs showed better performance, which was enhanced by *G. biloba* L. addition, due to the catalytic activity improving by cobalt and the additional active sites provided by *G. biloba* L. GNICPs reduced the abundance of bacteria, ARGs (*blaTEM*, *sul1*, *qnrA*, *acrA-02*, *mexB*, *tetM-01*, *ermB*, *mefA*, and *ereA*) and mobile genetic elements (*intI-1*, *intI3*, *tnpA-04*, and *TP614*). Notably, *acrA-02*, *blaTEM*, *ermB*, *mefA*, *mexB*, *qnrA*, and *tetM-01*, *intI3*, and *TP614* were reduced to below the detection limit. Another study evaluated the nZVI and nTiO_2_ NPs efficiency on the removal of the ARG *tetM*-carrying plasmids [144]. Both NPs were able to adsorb *tetM*-carrying plasmids; however, only nZVI could fragment the ARG by binding to PO_4_^3−^ of phosphate backbones, causing its disruption during desorption. 

Metallic NPs can also functionalize other materials to eliminate ARB and ARG from water. Jain and colleagues analyzed different coatings with metallic NPs (CuO, ZnO, or Ag_2_O) for water-resistant cellulose foam papers [148]. The coating with metallic NPs enhanced the filtration of analyzed microorganisms (*E. coli*, *P. aeruginosa*, *Bacillus Subtilis*, and *Bacillus cereus*). Moreover, foam papers coated with Ag_2_O NPs showed the best performance. Accordingly, melamine foams coated with Ag NPs used as filters completely removed *E. coli* bacteria from water, and no bacterial regrowth was registered. Synthesized Ag NPs from the pupa of green bottle fly were incorporated in a k-carrageenan film and showed great antimicrobial activity against different resistance bacteria belonging to the ESKAPE pathogens list, such as *P. aeruginosa*, *E. coli*, and *K. pneumoniae* [146].

Recently, another approach based on the electrocatalytic properties of carbon nanotubes coated on the titanium mesh by conductive agarose gel (CNTs/AG/Ti electrode) produced promising results concerning the inactivation of ARB and the degradation of ARGs in the aquatic environment [151]. Total inactivation of *E. coli* was achieved by using a potential of 1.8 V for 30 min that increased the production of reactive oxygen species and lead to bacteria cell membrane damage. In addition, the ARG *blaTEM-1* was also degraded after 30 min of electrocatalytic treatment due to DNA damage.

#### 4.2.2. ARBs and ARGs Removal by Microalgae-Based Technologies

Contrarily to antibiotics, only a few studies have addressed the removal of ARBs and ARGs by microalgae-based technologies [119,124,125] (Table 3). Rodrigues and colleagues reported that a microalgae-bacterial consortium was able to remove about 54% of sulfamethoxazole [125]. However, sulfonamide resistance gene *sul1* increased during the 7 days of the experiment. An explanation could be the selective pressure caused by the remaining sulfamethoxazole. On the other hand, another microalgae-bacteria consortium was able to reduce significantly *blaTEM* (72%) and *ermB* (97%) genes. Moreover, in this study, the antibiotics analyzed, cephalexin and erythromycin, were successfully eliminated (>90%) by the consortium [124]. These observations reinforce that the occurrence of antibiotics in water, even at low levels, produce a selective pressure, resulting in ARBs and ARGs dissemination. Taking this into account, and despite the evidence showing microalgae-bacteria consortium advantages, its applicability must be further explored to understand the mechanism and efficiency on the control of ARB and ARGs in water after the treatment.

## 5. Conclusions and Future Perspectives

In recent years, global efforts have been developed to raise awareness about antibiotic resistance including the EU Action Plan against antibiotic resistance (2011 and 2017), the World Health Organization Global Action Plan on antimicrobial resistance (2015), and the EU Guidelines for the prudent use of antimicrobials in human health [155]. Despite this, legislation regarding antibiotics monitorization on water sources and drinking water is still missing due to the lack of knowledge about their toxicity and occurrence in the environment. In 2015, the EU Commission published a watch list of substances to monitor in the environment, including three macrolides (erythromycin, clarithromycin, and azithromycin) [156]. Since then, this list has been reviewed in 2018 [157] and in 2020 [158], to include amoxicillin and ciprofloxacin, and sulfamethoxazole and trimethoprim, respectively. Besides the relevance of monitorization of antibiotics persistence on the environment and concretely on water sources, scientific evidence of ARG dissemination in water systems highlights this as an emergent public health concern that should be considered in global surveillance programs.

In addition, the ineffectiveness of conventional and advanced treatment processes in water treatment plants in the removal of antibiotics, ARB, and mainly ARGs, reinforces the demand to develop new or complementary methods. In this way, in recent years, a few studies have highlighted the benefits of nanotechnology and microalgae-based technologies’ application in water remediation. Nanomaterials and microalgae have shown remarkable performance considering the removal of antibiotics, which is crucial to reduce the selective pressure that largely contributes to ARB and ARG dissemination. Therefore, the complementary use of nanotechnology and microalgae with water treatment processes, such as reverse and forward osmosis, hold promises to achieve efficient removal of antibiotics and ARBs. Still, nanomaterials can be engineered to optimize their biocatalytic action and adsorption properties to inhibit antibiotic resistance dissemination.

The development of novel processes to eliminate ARGs in water treatment plants and in water distribution system remains a challenge, mainly because there are no safe levels of ARGs in water. Therefore, the reduction in the total or relative abundance of ARGs does not ensure the safety and quality of water to consume. Even at low levels, ARGs can be propagated among microorganisms and contribute to antibiotic resistance dissemination.

Thus, more research is required to develop these emergent technologies and upscale to achieve a proper elimination of ARGs in water treatment plants, but also along with the water distribution systems where the formation of biofilms promotes bacterial resistance and the dissemination of ARGs by horizontal gene transfer.

Another challenge is associated with the development of processes or systems that are environmentally friendly, such as biogenic NPs and microalgae-based technology, which also contribute to avoiding the release of by-products and toxicants for the environment. Moreover, these emergent and promising strategies overcome some important limitations and disadvantages of conventional methods, namely high cost of processing, energy consumption, and instability. So, it is critical to initiate scientific actions to develop these technologies with low environmental impact, to establish efficient, stable, scalable, and cost-effective solutions to the monitoring, control, and removal of these types of emergent pollutants from various aquatic systems.

## Figures and Tables

**Table 1:** ARGs removal efficiency by current water treatments.

Antibiotic Class	ARG	Water Treatment Process									
		CO + SE + SF + CL	SF	CL	OZ + CL	OZ	UV	BAC	GAC	MF	Ref
Aminoglycosides	*aadA*	↓			↓	↓	↑				[68]
	*aadB*	ND/↓						↑			[69]
	*aadE*	↓						↑			
	*aphA1*	ND/↑/↓									
	*strA*	↑/↓									[69,70]
	*strB*	↑/↓									
B-lactams	*bla_OXA-1_*	↓							↑		[70]
	*bla_CTX-M_*								↑		[71]
	*bla_TEM-1_*	↓		↓			↓		↑		[70,71,72]
Chloramphenicol	*cmlA*		↓			↑			↑		[73]
	*dfrA1*								↑		[71]
	*dfrA12*								↑		
Efflux pump	*opxB*		↓			↓			↑		[73]
	*mexF*	↑			↑	↑	↑				[68]
	*mexT*	↑			↑	↑	↑				
	*mexW*	↑			↑	↑	↑				
Florfenicol	*floR*		↓			↓			↑		[73]
Lincosamides	*cfr*		↓			↑			↑		
Macrolides	*ereA*	ND									[69]
	*ermB*	ND/↓ ↑	↓			↓			↑		[69] [70]
	*ermC*	↑									[70]
	*ermF*	ND								↓	[69,74,75]
	*ermG*	ND									
	*ermX*	ND									
	*mefA*	ND									
	*mph(A)*			↓			↓				[72]
Polypeptides	*bacA*	↓			↓	↓	↑				[68]
Quinolones	*qepA*	↓	ND						↑		[70,73]
	*qnrA*	↓	ND						↑		
	*qnrB*	↑	ND			↑			↑		
	*qnrD*		ND						↑		[73]
	*qnrS*		ND			↑			↑		
Sulfonamides	*sul1*	↓	↓	↓	↓	↓	↑/↓		↑	↓/↑	[68,70,71,72,73,74,75,76,77,78]
	*sul2*	↓	↓			↓			↑	↓/↑	[69,70,71,73,74,75,76,78]
	*sul3*	↓									[70]
Tetracyclines	*tetA*	↓	↓	↓		↑	↓	↑	↑	↑	[69,70,71,72,73,78]
	*tetB*	↑								↓	[70,74]
	*tetC*	↓									
	*tetM*	↑	↓			↑			↑	↓/↑	[70,73,75,78]
	*tetG*	ND							↑	↓	[69,71,74]
	*tetL*						↓				[79]
	*tetO*	ND/↓	↓			↓			↑	↑	[69,73,78]
	*tetQ*	ND	↓			↓			↑	↓	[69,71,73,75]
	*tetS*		↓						↑		[73]
	*tetW*	ND/↑	↓			↑			↑	↓/↑	[69,70,71,73,75,78]
	*tetX*	ND/↓	↓			↓			↑	↓/↑	[74,78]

BAC—biological activated carbon; CL—chlorination; CO—coagulation; GAC—granular activated carbon; ND—not detected; MF—membrane filtration; OZ—ozonation; SE—sedimentation; SF—sand filtration; UV—ultraviolet; ↑—increased expression; ↓—decreased expression.

**Table 2:** Recent promising strategies to efficiently eliminate antibiotics in wastewater and drinking water treatment plants.

Strategy	Target	Removal Efficiency	Ref
Nanomaterials (nanocomposites, nanofibers, NPs)			
TiO_2_-doped Fe^3+^ nano-photocatalyst	Metronidazole	97% (ci = 80 mg/mL, pH 11, 2 h) 69.85% (pH 6, 2 h)	[94]
Graphitized mesoporous carbon TiO_2_ nanocomposites	Ciprofloxacin	100% (ci = 1.5 mg/L, 1.5 h)	[95]
V_2_O_5_-ZnO NPs coated carbon nanofibers	Ciprofloxacin Cinoxacin	Adsorption of 87.70 mg/g (ci = 10–200 mg/L, pH 6.5, 20 min) Adsorption of 71.4 mg/g (ci = 10–200 mg/L, pH 6.5, 20 min)	[96]
Ta_3_N_5_ NPs/TiO_2_ hollow nanosphere composite	Levofloxacin Ciprofloxacin Tetracycline hydrochloride	93% (2 h); 89.76% after 4 cycles 93.2% (3 h) 92.2% (3 h)	[97]
Silver modified ZnO nanoplates	Ofloxacin	98% (ci = 10 mg/mL, pH 7, 2,30 h)	[98]
MnO_2_/graphene nanocomposite	Tetracycline	99.4%	[99]
SnO_2_/Ni@N carbon nanotubes	Cephalexin	>70% (electropersulfate oxidation)	[100]
Boron Nitride Nanosheets	Tetracycline Ofloxacin Cephalexin	Adsorption of 346.66 mg/g, pH 8 Adsorption of 72.50 mg/g (pH 8) Adsorption 225.0 mg/g (pH 12)	[101]
Green GS-NiFe beads nanocomposite	Tetracycline (ci = 20 mg/L)	Adsorption/degradation of 487 ± 6.85 mg/g Adsorption/degradation of 420 ± 10.21 mg/g Adsorption/degradation of 408 ± 12.35 mg/g	[102]
Green bimetallic nZVI-Cu NPs (pomegranate ring extract)	Tetracycline	72% (ci = 10 mg/L, pH 7)	[103]
Bentonite supported green nZVI-Cu nanocomposite		95% (pH 7)	
MnCo_2_O_4_ NPs	Ciprofloxacin	100% (pH 3, 5 h)	[104]
CdS NPs		79.50% (ci = 10 mg/mL, pH 9, 80 min)	[105]
NiFe_2_O_4_ NPs loaded graphitic carbon nitride	Oxytetracycline	100% (pH 5, 8 h)	[106]
ZV Cu (core) and Fe_3_O_4_ (shell) NPs	Oxytetracycline	>99% (ci = 20 mg/mL, pH3, 10 min)	[107]
S-doped MgO NPs	Tetracycline	90% (pH neutral, 10 min)	[108]
PRB columns packed with ZVI	Tetracycline (ci = 20 mg/L, pH 6.5, 30 days)	65%	[109]
PRB columns packed with MnO_2_		50%	
PRB columns packed with ZVI and MnO_2_		85% (pH 6.5, 30 days)	
Fe/Ni bimetallic NPs	Tetracycline	97.4% (ci = 100 mg/mL, pH 5, 3 h)	[110]
Microalgae			
*Microcystis aeruginosa*	Cefradine Amoxicillin	37.08% 60.89%	[111]
*Chlorella pyrenoidosa*	Cefradine Amoxicillin	42.63% 71.25%	
*Haematococcus pluvialis*	Sulfonamides	42–100% (mean 93%) of sulfamerazine, sulfamethoxazole, sulfamonomethoxine	[112]
*Selenastrum capricornutum*	Macrolides Fluoroquinolones	9–99% (mean 82%) of trimethoprim, clarithromycin azithromycin, roxithromycin 9–99% (mean 82%) of lomefloxacin, levofloxacin, flumequine	
*Scenedesmus quadricauda*	Sulfonamides	23–98% (mean 78%)	
*Chlorella vulgaris*	Macrolides Fluoroquinolones	10–100% (mean 47%) 10–100% (mean 47%) of fluoroquinolones (lomefloxacin, levofloxacin, flumequine)	
*Chlorella vulgaris*	Enrofloxacin Sulfadiazine Sulfamethazine Norfloxacin	53–73% 11–24% 16–33% Inefficient removal	[113]
*Chrysosporum ovalisporum*	Enrofloxacin Sulfadiazine Sulfamethazine Norfloxacin	58–79% 10–20% 14–27% Inefficient removal	
*Chlorella pyrenoidosa*	cefuroxime sodium	60% (within 48 h) 92.9% (with NaHCO3 addition)	[114]
*Scenedesmus obliquus*	Ofloxacin	9.95–39.24%	[115]
*Scenedesmus dimorphus*	Ofloxacin	93%	[116]
*Spirulina* sp.-derived biochar	Tetracycline	Adsorption of 61% (120 h; ↓ adsorption along with cycles)	[117]
*Scenedesmus obliquus*	Sulfamethazine Sulfamethoxazole	31.4–62.3% (12 days) 27.7–46.8% (12 days)	[118]
*Chlorella micrococcus* (photo-sequencing batch reactor)	Trimethoprim Sulfamethoxazole Sulfamethazine Sulfamerazine Norfloxacin Enrofloxacin	91.8% 85.5% 85.5% 85.5% 98% 100%	[119]
*Chlorella pyrenoidosa*	Sulfamethoxazole	Biodegradation of 14.9% (11 days) 99.3% (with sodium acetate addition, 5 days)	[120]
*Chlorella vulgaris* (batch culture)	Sulfadiazine Sulfamethazine Sulfamethoxazole	32.06% (12 days) 31.17% (12 days) 34.07% (12 days)	[121]
*Chlorella vulgaris* biofilm membrane photobioreactor	Sulfadiazine Sulfamethazine Sulfamethoxazole	79.2% (1 day) 76.7% (1 day) 82.1% (1 day)	
*M. aeruginosa*	Tetracycline	98% (2 days)	[122]
*Chlamydomonas* sp. Tai-03	Ciprofloxacin Sulfadiazine	100% (65.05% by biodegradation) 54.53% (35.60% by photolysis)	[123]
Microalgae-bacteria consortium	Cephalexin Erythromycin	96.54% (7 days) 92.38% (7 days)	[124]
	Sulfamethoxazole	54.34% (42.86% by biodegradation)	[125]

NPs—nanoparticles; PRB—permeable reactive barrier; and ZVI—zero-valent iron.

**Table 3:** Promising strategies to eliminate ARBs and ARGs in water treatment plants and drinking water distribution systems.

Strategy	Result	Ref
Nanotechnology (nanoparticles, nanocomposites, nanofibers)		
GNICPs (Ginkgo biloba L. modified iron-cobalt NPs)	↓ bacterial abundance (↓16S rRNA) ↓ ARGs: *bla_TEM_*, *sul1*, *qnrA*, *acrA-02*, *mexB*, *tetM-01*, *ermB*, *mefA*, *ereA* ↓MGEs: *intI1*, *intI3*, *tnp-04*, and *TP614* Altered microbial community composition	[147]
Cd^2+^ and Fe_2_O_3_ NPs	↑ conjugative transfer frequencies ↑ cell membrane permeability ↑ antioxidant enzymes (SOD, CAT) ↑ mRNA expression of *trfAp* and *trfBp*	[152]
Metallic (Cu, Zn, CuO, ZnO) NPs	↓ bacterial growth ↓ ARGs: *sul1*, *aadA1* and MGE: *intl1* ↑ ROS production ↑ bacterial cell membrane permeability	[143]
nTiO_2_ NPs	Adsorption of *tetM*-plasmid (0.06/min and 4.29 mg/g)	[144]
nZVI NPs	Adsorption of *tetM*-plasmid (0.05/min and 2.15 mg/g) ARGs fragmentation	
CuO NPs (with humic acid)	↓ absolute ARGs: *macB*, *mexF* and MGE: *intl1* ↓ absolute metallic-resistance genes: copA, cusA Modulation of EPS production	[145]
CNTs/AG/Ti electrode (Carbon nanotubes/agarose/titanium)	↑ ROS production bacterial cell membrane damage ↓ ampicilin-resistant *E. coli* (100%, 1.8.V, 30 min) *bla_TEM-1_* degradation (100%, 2 V (PBS), 30 min)	[151]
Water-resistant cellulose foam paper coated with CuO, ZnO, or Ag_2_O NPs	Enhanced cellulose filter paper antibacterial activity against *E. coli*, *P. aeruginosa*, *Bacillus Subtilis*, and *Bacillus cereus* Ag_2_O NPs produced the highest antibacterial activity	[148]
Melamine foams with Ag NPs	Antibacterial activity against *E. coli*	[149]
PVDF membrane functionalized with TiO_2_ NPs	99.9% retention of tetracycline, chloramphenicol, and sulfadiazine-resistant bacteria ↓ ARGs: *floR* (97.8%), *sul1* (99.5%), *sul2* (98.8%), *intI1* (93.7%), *tetC* (20.6%), *tetW* (27.2%), *tetQ* (2.0%) Inhibition of HGT	[150]
Chitosan/biochar-nanosilver (C-Ag) composite	Sustainable antibacterial activity against *E. coli* (>50 days) Good reusability	[153]
Carbon-based copper nanocomposites	↓ absolute ARGs and MGEs ↓ HGT mediated by plasmids and MGEs	[142]
SWNTs-PAN/TPU/PANI composite electrospun nanofiber membrane	Complete removal of *S. aureus* and *E. coli* Good durability and stability over various cycles	[154]
k-carrageenan/Ag NPs film	Antimicrobial activity against *Vibrio cholerae*, *Candida albicans*, *P. aeruginosa*, *E. coli*, *K. pneumoniae*, and *Bacillus cereus* Inhibition of bacteria growth	[146]
Microalgae		
*Chlorella* micrococcus (photo-sequencing batch reactor)	↓ 78% ARG absolute abundance ↓ *Rhodocyclaceae* and *Burkholderiaceae* bacteria families	[119]
Microalgae-bacteria consortium	↓ ARGs: *bla_TEM_* (72%) and *ermB* (97%) absolute abundance	[124]
	↑ ARG: *sul1*	[125]

ARG—antibiotic resistance gene; CAT—catalase; EPS—extracellular polymeric substances; HGT—horizontal gene transference; NPs—nanoparticles; MGE—mobile genetic element; PRB—permeable reactive barrier; ROS—reactive oxygen species; SOD—superoxide dismutase; ZVI—zero-valent iron; ↓—decrease; and ↑—increase.
